# Compared to casein, bovine lactoferrin reduces plasma leptin and corticosterone and affects hypothalamic gene expression without altering weight gain or fat mass in high fat diet fed C57/BL6J mice

**DOI:** 10.1186/s12986-015-0049-7

**Published:** 2015-12-08

**Authors:** Bettina McManus, Riitta Korpela, Paula O’Connor, Harriet Schellekens, John F. Cryan, Paul D. Cotter, Kanishka N. Nilaweera

**Affiliations:** Teagasc, Moorepark Food Research Centre, Fermoy, County Cork, Ireland; Faculty of Medicine, Pharmacology, Medical Nutrition Physiology, University of Helsinki, Helsinki, Finland; Alimentary Pharmabiotic Centre, University College Cork, Cork, Ireland; Department of Anatomy & Neuroscience, University College Cork, Cork, Ireland

**Keywords:** Lactoferrin, High fat diet, Weight gain, Fat mass, Obesity, Hypothalamus, Microarray, Leptin, Corticosterone

## Abstract

**Background:**

Several studies in both humans and rodents have examined the use of lactoferrin as a dietary solution to weight gain and visceral fat accretion and have shown promising results in the short term (up to 7 weeks). This study examined the effects of giving lactoferrin over a longer period of time.

**Methods:**

For 13 weeks, male C57/BL6J mice were given a diet containing 10 % kJ fat and 20 % kJ casein (LFD) or a diet with 45 % kJ fat and either 20 % kJ casein (HFD) or 20 % kJ lactoferrin (HFD + Lac). Physiological, metabolic, and biochemical parameters were investigated. Gene expression was investigated by Real-Time PCR and microarray. All data was assessed using t-test, ANOVA or ANCOVA. Gene Set Enrichment Analysis was used to interpret microarray data and assess the impact on gene sets with common biological roles.

**Results:**

By the end of the trial, HFD + Lac fed mice did not alter energy balance, body composition, bodyweight, or weight gain when compared to the HFD group. Notably, there were no changes in subcutaneous or epididymal adipose leptin mRNA levels between high fat diet groups, however plasma leptin was significantly reduced in the HFD + Lac compared to HFD group (*P* < 0.05) suggesting reduced leptin secretion. Global microarray analysis of the hypothalamus indicate an overall reduction in gene sets associated with feeding behaviour (*P* < 0.01) and an up-regulation of gene sets associated with retinol metabolism in the HFD + Lac group compared to the HFD group (*P* < 0.01). Genes in the latter catergory have been shown to impact on the hypothalamic-pituitary-adrenal axis. Notably, plasma corticosterone levels in the HFD + Lac group were reduced compared to the HFD fed mice (*P* < 0.05).

**Conclusions:**

The data suggests that prolonged feeding of full-length dietary lactoferrin, as part of a high fat diet, does not have a beneficial impact on weight gain when compared to casein. However, its impact on leptin secretion and accompanying changes in hypothalamic gene expression may underlie how this dietary protein alters plasma corticosterone. The lactoferrin fed mouse model could be used to identify leptin and corticosterone regulated genes in the hypothalamus without the confounding effects of body weight change.

**Electronic supplementary material:**

The online version of this article (doi:10.1186/s12986-015-0049-7) contains supplementary material, which is available to authorized users.

## Background

The number of people who can be classified as overweight (body mass index = 25–29.9 kg/m^2^) or obese (body mass index ≥ 30 kg/ m^2^) is expected to rise to include as much as 75 % of the population in the United States and up to 70 % in the UK by the year 2020 [1]. Health implications associated with obesity, such as certain types of cancer, cardiovascular disease, diabetes mellitus type 2, and non-alcoholic fatty liver disease, reduce life expectancy and quality of life and at the same time increase the medical costs required to care for those afflicted with such diseases [1]. As increases in obesity have such an impact on the individual and society as a whole, greater research into dietary solutions for obesity has been undertaken in recent years.

One such dietary solution is focused on the use of whey protein isolate (WPI), which has been shown to reduce weight gain associated with the intake of high fat diets [2, 3]. Tranberg et al*.* showed that the beneficial impact WPI has on weight gain in high fat diet fed adolescent mice took place primarily during the initial four weeks of WPI intervention when mice were 5 to 9 weeks old [3]. Following this time point, high fat diet-induced weight gain is re-established, however, final body weight reductions are still significant due to the early effects of WPI intervention on weight gain [3]. Previously, we found that bovine serum albumin (BSA), a constituent protein of WPI, had a significant impact on weight gain in mice when diet intervention was started at a later stage in adolescence than those in the Tranberg et al*.* study [4]. In our BSA study, 8 week old male mice were provided with high fat diets containing either casein or BSA as the dominant source of protein [4]. After 13 weeks of high fat diet feeding, BSA fed mice had significantly reduced body weight compared to the casein control, similar to the WPI fed mice in the Tranberg et al*.* study [3, 4]. In contrast to the Tranberg et al*. study*, BSA fed mice reduced weight gain from 8 to 16 weeks of age during the transition from adolescence to adulthood, which suggests an impact of BSA on weight gain for a lengthier period of time and across a broader age range than WPI [4].

Lactoferrin, a glycoprotein found in whey, has been shown to have beneficial effects in the management obesity over multiple age categories [5]. For instance, in comparison to mice given casein, mice given bovine lactoferrin as a supplement to casein (15 % bovine lactoferrin: 85 % casein) have been shown to increase weight loss on a 70 %-reduced calorie-restricted diet for 50 days and impede weight regain when given *ad libitum* access to food over the course of another 50 days [5]. Similarly, gastric intubation of bovine lactoferrin in 8-week old ICR mice fed a standard diet for 4 weeks resulted in reduced visceral adipose mass and hepatic triacylglycerol (TAG) accumulation [6]. In a different trial carried out by Takeuchi et al*.,* ICR mice fed a standard chow diet and supplemented with bovine lactoferrin over 4 weeks had significantly reduced plasma non-esterified fatty acids (NEFA), TAG, and increased high-density lipoprotein cholesterol; however, mice fed a high fat diet and supplemented with bovine lactoferrin showed no change in these lipid metabolites compared to the control high fat diet [7]. Likewise, Sprague–Dawley rats fed a high fat diet with gastric intubation of bovine lactoferrin did not have any observed beneficial impact compared to controls [6]. In light of the inconsistent results, further work is required to understand the effects of bovine lactoferrin on weight gain when given alongside a high fat diet over a prolong period of time.

To this end, male C57BL/6 J mice were provided with high fat diets containing either casein or bovine lactoferrin at 8 weeks of age, which corresponded with our BSA study as well as the Morishita et al*.* study [4, 6]. Diets continued for 13 weeks, in line with our BSA trial, to examine the impact of bovine lactoferrin on energy balance over a longer duration during the growth period between adolescence to fully mature adult mice. As part of this study, we undertook a microarray approach to assess the impact of lactoferrin on the hypothalamic gene expression important for energy balance regulation and subjected the data to a powerful analytical method called Gene Set Enrichment Analysis (GSEA), as detailed by Subramanian et al*.* [8]. Rather than focusing on individual genes, GSEA approach assesses if gene sets with common biological roles or regulation are affected by the intervention of interest. This provides a more meaningful answer to how an intervention affected physiological processes by focusing on pathways rather than individual genes and gave greater insight into how lactoferrin affects key energy balance related parameters in HFD fed mice.

## Methods

### Animals and diets

The animals in this experiment were maintained in accordance with a license obtained under the Cruelty to Animals Act 1876, and all work adhered with University College Cork Animal Ethics Committee approval (#2011/005). Male C57/BL6J mice, aged 3–4 weeks (Harlan, Middlesex, UK), were weight matched (17.89 ± 1.68 g) and randomly group housed (4 per cage, 8 per group) in individually ventilated cages. The environment animals were kept in was 20–22 °C, 45–65 % humidity, and in 12:12 h light–dark cycles. Mice had *ad libitum* access to fresh water and one of three diets (Research Diets, New Brunswick, USA): LFD (low fat diet; 10 % energy fat and 20 % energy casein; D12450B), HFD (high fat diet with casein; 45 % energy fat and 20 % energy casein; D12451), or HFD + Lac (high fat diet with bovine lactoferrin; D12451 with 20 % energy bovine lactoferrin; Glanbia, Carlsbad, USA), throughout the study. Details on the diets are in Additional file 1: Table S1.

### Experimental protocol

During the 5-week acclimatization period, mice were allowed *ad libitum* access to the same LFD and fresh water. Subsequent to acclimatization, mice were weight matched and provided with one of 3 diets: LFD, HFD, or HFD + Lac (*N* = 8). Weekly bodyweight and food intake measurements were recorded. To measure metabolic parameters, mice were housed individually for 72 h in TSE PhenoMaster system cages (TSE systems, Bad Homburg, DE) during weeks 9–11 before returning to their home cages. In week 13, immediately prior to termination, body composition was determined using a Bruker Minispec LF50H (Bruker Optics, Stillorgan, EI). Mice were fasted for 4–6 h, anesthetized (65 mg/kg ketamine and 13 mg/kg xylazine), and blood was collected into vacutainer EDTA tubes (Becton, Dickinson and Company, Dublin, EI). Upon collection, blood was mixed with 500,000 KUI/L Aprotinin (Sigma-Aldrich, Cambridge, UK) and 0.1 mM Diprotin A (Sigma-Aldrich, Cambridge, UK). Cervical dislocation was used to euthanize mice, and tissues were harvested, weighed, and snap frozen in dry ice (brain) or liquid nitrogen before being stored at −80 °C.

### Metabolic parameters

The TSE Phenomaster system, comprised of 8 test cages and 1 reference cage, were calibrated such that feeding sensors measured food consumption above 0.01 g, and collected data every 13 min (1.5 min per cage) over the final 24 h of the 72 h housing period. Indirect open-circuit calorimetry was used to measure carbon dioxide (VCO_2_) production and oxygen (VO_2_) consumption, and the respiratory exchange ratio (RER = VCO_2_/VO_2_) was calculated using this data. The sensor and time recording procedures follow those set forth by McAllan, et al. except that locomotor activity included the Z-axis in addition to the X and Y axes [2, 9].

### Gene expression

Two approaches were used; Real-Time (RT)-PCR and microarray. The RNA extraction and RT-PCR protocols, including product and approach, followed protocols set forth by McAllan et al*.* [2]. A geometric mean was determined in instances where two or more housekeeping genes were used for normalization [10]. Reference genes included: tyrosine 3-monooxygenase/tryptophan 5-monooxygenase activation protein, zeta polypeptide (*Ywhaz*) and β-actin (*Actb*) in the jejunum, 18S ribosomal 1 (*Rna18s*) and *Actb* in adipose tissue, and *Rna18s* and *Ywhaz* in the hypothalamus [2, 9, 11]. The Pfaffl method was used to determine gene expression relative to LFD controls [12]. Primer sequences can be found in Additional file 2: Table S2.

The hypothalamic gene expression was also investigated using the microarray approach in mice from the HFD and HFD + Lac groups (*N* = 3). RNA quality was established on an Agilent Bioanalyzer 2100 using the 2100 expert Eukaryote Total RNA Nano series II software (Agilent Technologies, Espoo, FI). Once quality and concentration were determined, 200 ng of RNA was converted to cDNA using AffinityScript RNase Block and labelled according to manufacturer’s instructions (Agilent Technologies, Espoo, FI). Amplified and labelled samples were then purified in Qiagen RNA mini tube columns (Qiagen, Helsinki, FI) and the success of labelling was determined using an RNA Spike-In Kit per manufacturer’s protocol (Exiqon, Vedbaek, DK). Samples were then mixed with blocking agent, hybridization and fragmentation buffer, and hybridized to Agilent SurePrint G3 Mouse GE 8 × 60 K microarrays at 65 °C for 17 h before they were washed and scanned using an Agilent Scanner G2505C as set out in the manufacturer’s directions (Agilent Technologies, Espoo, FI).

### Western blot

All procedures pertaining to protein extraction from adipose tissue, SDS-PAGE, Western blotting, and blocking were performed according to previously defined methods with some modifications [2]. Specifically, membranes were incubated overnight at 4 °C with the primary antibody against peroxisome proliferator-activated receptor alpha (PPARA) (1:500, H-98 SC-9000, Santa Cruz Biotechnology, Dublin, EI) and against ccaat-enhancer binding protein alpha (CEBPA) (1:500, 14AA SC-61, Santa Cruz Biotechnology, Dublin, EI). HRP-conjugated donkey anti-rabbit secondary antibodies (1:7500 dilution; 711-035-152, Jackson Immuoresearch, Newmarket, UK) were incubated at room temperature for 60 min. Membranes were stained with a 1:15000 dilution of a β-actin-HRP antibody (A3854, Sigma-Aldrich, Cambridge, UK) to correct for sample loading. ECL Western blotting Substrate Kit (Thermo Scientific, Dublin, EI) was used for visualization.

### Plasma metabolites and hormones

Commercially available ELISA or calorimetric kits were used to measure corticosterone, leptin, insulin, glucose, and TAG levels (Corticosterone: Enzo Life Sciences, Exeter, UK; leptin and insulin: Alpha Diagnostics, Eastleigh, UK; glucose: Abcam, Cambridge, UK; TAG: Wako, Cambridge, USA). Insulin sensitivity in each group was determined by calculating QUICKI from data collected from plasma samples (1/(log [insulin (μU/L)] + log [glucose (mg/dL])) [13].

To measure plasma amino acid levels in the high fat diet groups, samples were deproteinated with 24 % (^w^/_v_) trichloroacetic acid at a ratio of 1:1, incubated for 10 min at room temperature, and centrifuged at 14400 rpm (Microcentaur, MSE, UK) for 10 min. Next, the supernatant was diluted with the internal standard norleucine, and the quantification of amino acids was completed using a Joel JLC-500/V amino acid analyzer (Joel Ltd., Garden City, Herts, UK) fitted with a Joel Na^+^ high performance cation exchange column.

### Fecal fat analysis

A modified version of the Folch method was used to determine % fecal fat [14, 15]. First, pellets were thawed, distilled water was added (10 mL to 1 g), and they were allowed to rest at room temperature overnight. Next, chloroform-methanol (2:1 ^v^/_v_) and 0.58 % (^w^/_v_) aqueous NaCl solutions were mixed with samples using a vortex prior to centrifugation. Then the chloroform phase was measured and transferred to pre-weighed tubes before evaporation. Finally, the tubes were re-weighed to determine total lipid content in the fecal mass. All analyses were carried out in duplicate.

### Statistics

Statistical analyses were completed with the aid of Prism (GraphPad Software, Inc., La Jolla, USA) and Minitab (Minitab Inc., Coventry, UK). ANOVA with a Tukey post hoc test was used to compare 3 groups in all analyses of results except oxygen consumption (VO_2_) where an ANCOVA (SAS software version 9.3, Cary, USA) with total body weight as the co-variant was used. To analyze Western blot data, non-parametric multiple comparisons Kruskal-Wallis test followed by, where appropriate, Mann–Whitney U tests for individual comparisons were used. The significance of results was established at *P* < 0.05 and reported as mean ± SEM.

For the microarray analysis, background was corrected with the normexp method, normalized, and log 2-tranformed before differences between high fat diet groups were determined using Bayes moderated t-statistics. Gene set enrichment analysis of the microarray data compared significant similarities between differential expression of HFD and HFD + Lac groups against known gene sets in the Molecular Signatures Database Version 5.0 (MSigDB), and normalized data was run through the GSEA.

## Results

### Dietary bovine lactoferrin did not alter weight gain or body composition but did reduce fecal fat excretion compared to casein

**Fig. 1:**
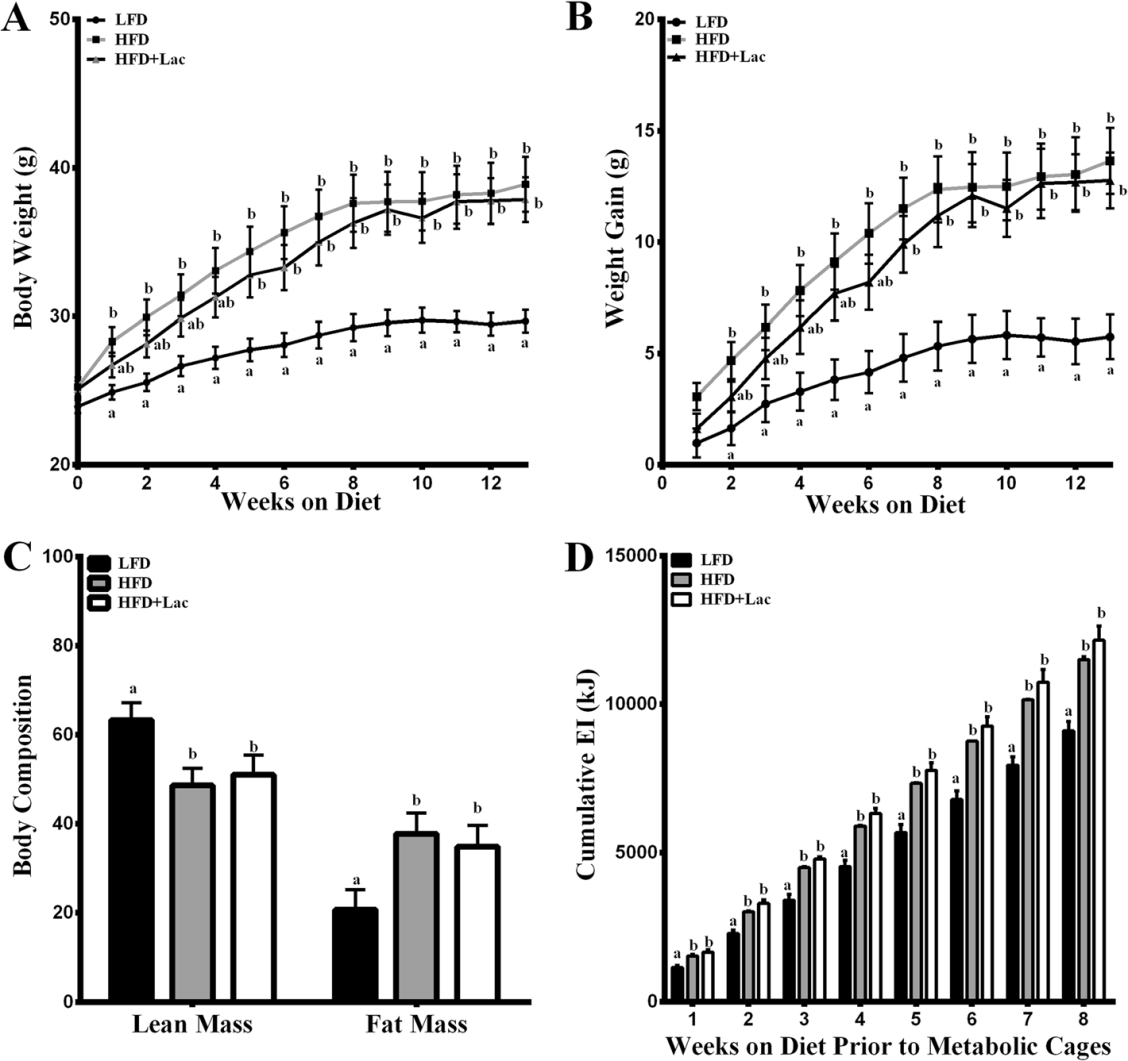
Effect of dietary bovine lactoferrin on (**a**) bodyweight, (**b**) weight gain, (**c**) body composition, over a 13 week period, and (**d**) cumulative energy intake (kJ) through week 8 in C57/BL6 mice. Data represent mean values ± SEM (*n* = 8). Different letters indicate a significant difference *P* < 0.05. Abbreviations: LFD, 10 % energy fat diet with 20 % energy casein; HFD, 45 % energy fat diet with 20 % energy casein; HFD + Lac, 45 % energy fat diet with 20 % energy lactoferrin

Initially, there was a 4 week delay in achieving a significant bodyweight increase in HFD + Lac fed mice compared to the LFD not seen in the HFD group, which was significantly increased in week 1 (Fig. 1a). Likewise, while the HFD group achieved a significant increase in weight gain compared to the LFD group by week 2, the HFD + Lac group did not reach a significant increase in weight gain over the LFD group until week 8 (Fig. 1b). By the end of the 13 week trial, both high fat diet groups were significantly greater in % fat mass and reduced in % lean mass in comparison to the LFD controls (Fig. 1c). In support of body fat mass percentages, SAT and EAT fat pads collected from the HFD and HFD + Lac groups had similar masses and were both significantly greater than in the LFD group (Table 1). Analysis of metabolic cage data collected during weeks 9–12 indicated no changes in EI between groups (Table 1); however, cumulative EI over the length of the study was greater in both high fat diet groups when compared to the LFD mice (Fig. 1d). When using bodyweight as a co-variant, a trend toward decrease in VO_2_ was noted in the HFD + Lac group compared to the LFD group (*P* = 0.08) (Table 1). The respiratory exchange ratio, consistent with fat as a primary fuel source, was significantly reduced in both high fat diet fed groups compared to the LFD (Table 1), and there was no change in locomotor activity between all three groups (Table 1).

**Table 1:** Metabolic parameter assessments over 24 h for mice fed for 13 weeks with either a 10 % kJ low fat diet with 20 % casein (LFD), 40 % kJ fat diet with 20 % casein (HFD), or 40 % kJ fat diet 20 % lactoferrin (Lac)

	LFD	HFD	Lac
Energy Intake (kJ)	35.37 ± 2.94	41.11 ± 3.91	50.18 ± 5.25
VO_2_ (ml/min)	1.81 ± 0.03	1.78 ± 0.03	1.71 ± 0.03
Locomotor Activity (Beam breaks)	229.79 ± 9.75	200.12 ± 12.47	196.35 ± 18.90
RER	0.82 ± 0.02^a^	0.75 ± 0.01^b^	0.76 ± 0.01^b^
Insulin (pmol/L)	5.62 ± 0.10	6.74 ± 0.26	6.61 ± 0.59
Glucose (mmol/L)	10.00 ± 0.62	12.01 ± 1.09	11.01 ± 0.99
QUICKI	0.46 ± 0.01	0.44 ± 0.01	0.45 ± 0.00
FFA (nmol/mL)	0.19 ± 0.04	0.46 ± 0.04	0.36 ± 0.07
SAT mass (g)	0.30 ± 0.02	0.95 ± 0.12	0.77 ± 0.10
EAT mass (g)	0.83 ± 0.10	1.65 ± 0.14	1.79 ± 0.26

All data is represented as mean ± SEM (*N* = 8). Dissimilar letters represent significance (*P* < 0.05)

**Fig. 2:**
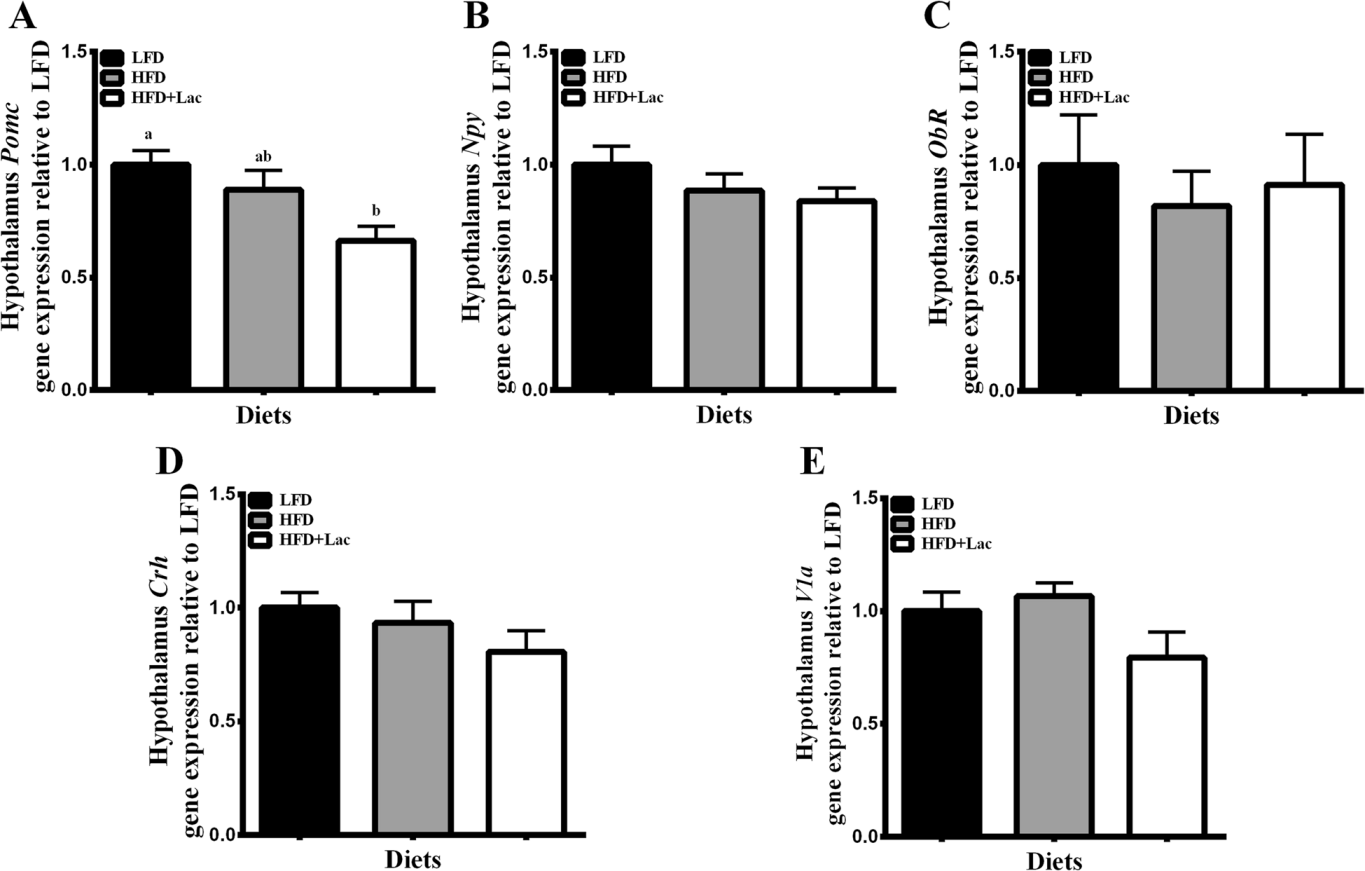
Effect of dietary bovine lactoferrin on hypothalamic gene expression of pro-opiomelanocortin (*Pomc*) (**a**), neuropeptide Y (*Npy*) (**b**), leptin receptor (*Obr*) (**c**), corticotropin releasing hormone (*Crh*) (**d**), and arginine vasopressin receptor 1a (*V1a*) (**e**). Data is represented as mean values ± SEM (*n* = 8). Different letters indicate significant differences, and significance is determined at *P* < 0.05. Abbreviations: LFD, 10 % energy fat diet with 20 % energy casein; HFD, 45 % energy fat diet with 20 % energy casein; HFD + Lac, 45 % energy fat diet with 20 % energy lactoferrin

Regarding hypothalamic mRNA levels of genes associated with the regulation of EI and energy expenditure, pro-opiomelanocortin (*Pomc*) was significantly decreased in the HFD + Lac group when compared to the LFD group (Fig. 2a), however, neither neuropeptide Y (*Npy*) nor leptin receptor (*Ob-r*) were significantly altered between groups (Fig. 2b-c). Interestingly, when microarray analysis of a gene-set associated with leptin and feeding behavior was analyzed, there was a significant decrease in this gene set in the HFD + Lac group compared to the HFD group (Table 2).

**Table 2:** Hypothalamic gene-set analysis comparing the HFD and HFD + Lac groups^a^

Name	Systematic name	NES	NOM *p*-value	FDR *q*-value
Feeding behavior	M17480	−1.7317742	*P* < 0.01	0.09125667
*Agrp*				
*Brs3*				
*Cartpt*				
*Cckar*				
*Cckbr*				
*Fyn*				
*Galr2*				
*Glg*				
*Ghr1*				
*Ghsr*				
*Hctrl*				
*Mc4r*				
*Pmch*				
*Pyy*				
*Ubr3*				
Kegg retinol metabolism	M9488	1.8021116	*P* < 0.01	0.016435869
*Rdh12*				
*Cyp26a1*				
*Aldh1a1*				
*Rdh8*				
*Rdh11*				
*Retsat*				
*Cyp26b1*				
*Rpe65*				
*Dgat2*				
*Aldh1a2*				
*Dhrs3*				

^a^ Gene names: *Agrp,* agouti related protein homolog (mouse); *Brs3*, bombesin-like receptor 3; *Cartpt*, CART prepropeptide, *Cckar*, cholecystokinin A receptor; *Cckabr*, cholecystokinin B receptor; *Fyn*, FYN oncogene related to SRC, FGR, YES; *Galr2*, galanin receptor 2; *Gcg*, glucagon; *Ghrl*, ghrelin/obestatin; *Ghsr*, ghrelin/obestatin prepropeptide; *Hcrtr1*, hypocretin (orexin) receptor 1; *Mc4r*, melanocortin 4 receptor; *Pmch*, pro-melanin-concentrating hormone; *Pyy*, peptide YY; *Ubr3*, ubiquitin protein ligase E3 component n-recognin 3; *Rdh12*, retinol dehydrogenase 12 (all-trans/9-cis/11-cis); *Cyp26a1*, cytochrome p450, family 26, subfamily A, polypeptide 1; *Aldh1a1*, aldehyde dehydrogenase 1 family, member A1; *Rdh*, retinol dehydrogenase 8 (all-trans); *Rdh11*, retinol dehydrogenase 11 (all-trans/9-cis/11-cis); *Retsat*, retinol saturase (all-trans-retinol 13, 14-reductase); *Cyp26b1*, cytochrome p450, family 26, subfamily B, polypeptide 1; *Rpe65*, retinal pigment epithelium-specific protein 65 kDa; *Dgat2*, diacylglycerol O-acyltransferase 2; *Aldh1a2*, Aldehyde dehydrogenase 1 family, member A2; *Dhrs3*, dehydrogenase/reductase (SDR family) member 3

Abbreviations: *NES* normalized enrichment score; *NOM* nominal; *FDR* false discovery rate

**Fig. 3:**
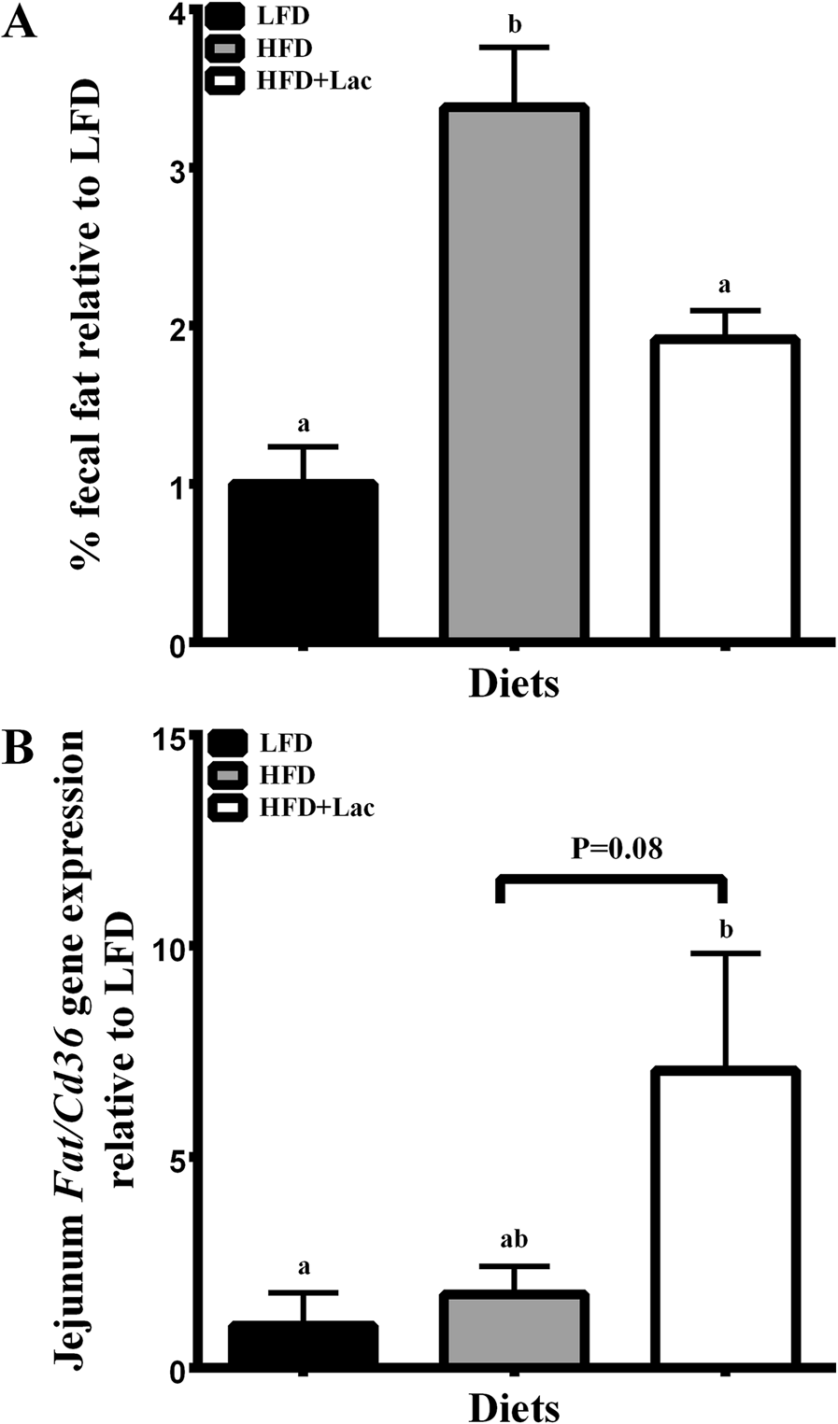
Effect of dietary bovine lactoferrin on % fecal fat (**a**) and jejunal mRNA levels of Fatty acid transporter *(Cd36/FAT)* (**b**) taken from male C57/BL6J mice after 13 weeks of experimental diet feeding. Data is represented as mean values ± SEM (*n* = 8). Different letters indicate significant differences, and significance is determined at *P* < 0.05

Lipid excretion was examined in fecal samples and found to be significantly less in the LFD and HFD + Lac groups when compared to the HFD control group (Fig. 3a), which is curious given the inherent difference in fat content between the low fat and high fat diets. To explore this result further, jejunal mRNA levels associated with fatty acid transporter (*FAT/Cd36),* were examined, and it was found that they were significantly increased in the HFD + Lac group compared to the LFD mice (*P* < 0.05) and also showed an increased trend compared to the HFD group (Fig. 3b).

### Plasma leptin and corticosterone were significantly reduced in mice fed dietary bovine lactoferrin compared to casein in a high fat diet

**Fig. 4:**
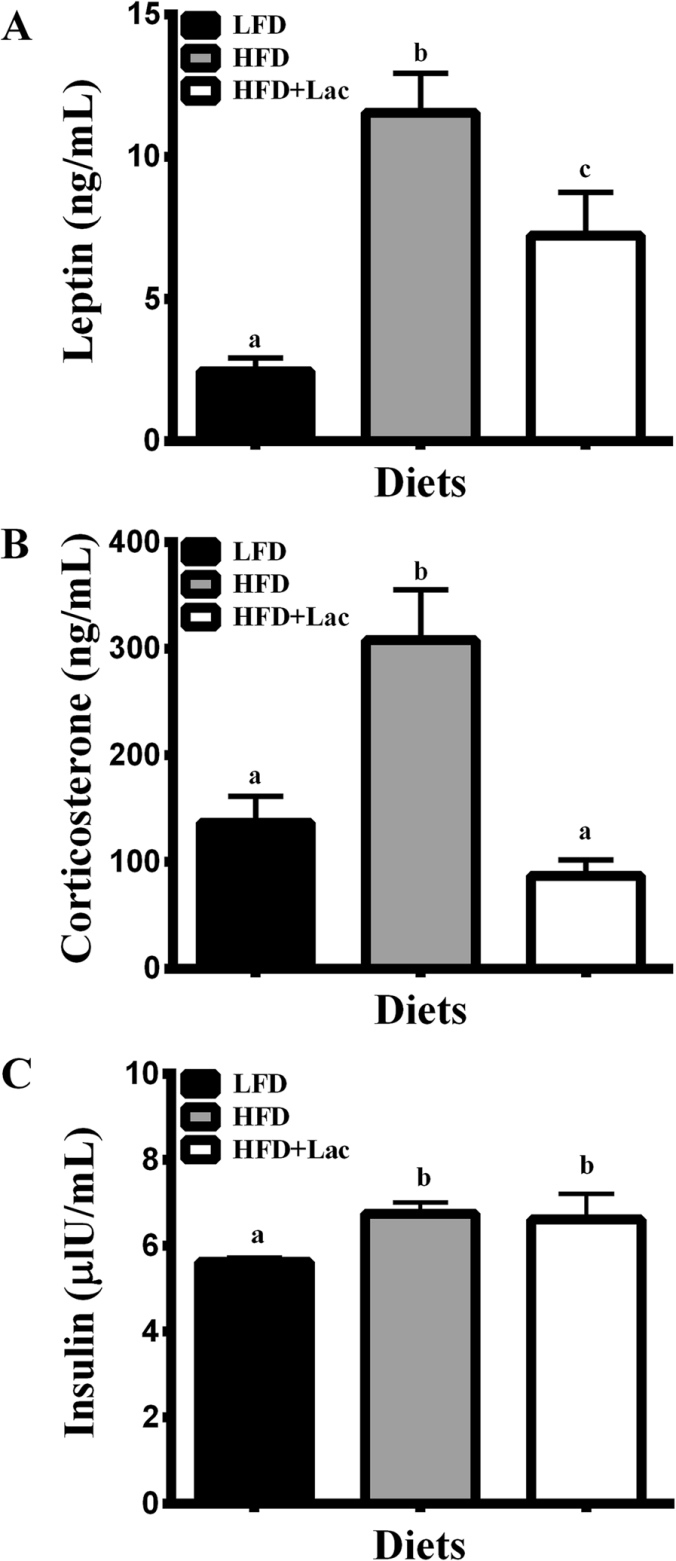
Effect of dietary bovine lactoferrin on plasma leptin (**a**), corticosterone (**b**), and insulin (**c**) concentrations taken from male C57/BL6J mice after 13 weeks of experimental diet feeding. Data is represented as mean values ± SEM (*n* = 8). Different letters indicate significant differences, and significance is determined at *P* < 0.05. Abbreviations: SAT, subcutaneous adipose tissue; LFD, 10 % energy fat diet with 20 % energy casein; HFD, 45 % energy fat diet with 20 % energy casein; HFD + Lac, 45 % energy fat diet with 20 % energy lactoferrin

Despite similar % fat mass to the HFD group, HFD + Lac fed mice had significantly reduced circulating leptin concentrations (Fig. 4a). Likewise, plasma corticosterone levels were significantly less in the bovine lactoferrin group than in the HFD group (Fig. 4b). In the hypothalamus, mRNA expression of genes associated with central control of corticosterone production, namely corticotropin releasing hormone (*Crh*) and arginine vasopressin receptor 1a (*V1a*) did not change between groups (Fig. 2d-e), however the Kegg Retinol Metabolism pathway showed a significant increase in the HFD + Lac group compared to the HFD fed mice (Table 2).

To ascertain an underlying mechanism for how the plasma leptin changes in lactoferrin fed mice, we compared the plasma amino acid concentrations in high fat diet groups and found that mice fed lactoferrin had significantly reduced histidine (*P* < 0.01) and glutamate (*P* < 0.01) and increased glycine (*P* < 0.05) and isoleucine (*P* < 0.05) concentrations while other amino acids remained unchanged between groups (Table 3). Plasma insulin concentrations were significantly greater in both high fat diet fed groups compared to the LFD mice (Fig. 4c), however, there were no significant changes in plasma glucose or QUICKI levels (Table 1). It is worth mentioning that plasma TAG concentrations were significantly increased in the HFD + Lac group compared to the LFD group, but no such increase was observed in the HFD group (Table 1).

**Table 3:** Plasma amino acid concentrations in mice fed a HFD or HFD + Lac for 13 weeks were determined using Joel Na^+^ high performance cation exchange column^c^

Amino acids	HFD		HFD + Lac	
	Mean	SEM	Mean	SEM
Glu	89.40^a^	3.68	69.10^b^	3.38
Gly	135.00^a^	4.27	164.00^b^	8.38
His	103.00^a^	8.79	67.02^b^	2.35
Ile	205.00^a^	9.87	267.00^b^	25.10
Asp	7.58	0.96	8.54	1.42
Cys 1 + 2	23.80	1.70	20.40	0.51
Leu	78.80	3.89	72.60	7.68
Val	131.00	5.85	111.00	6.88
Arg	51.30	8.57	61.30	9.10
Ser	65.01	2.43	67.90	5.19
Pro	113.00	20.30	169.00	28.50
Tyr	43.10	4.41	39.50	1.88
Phe	36.60	1.88	36.60	2.15
Lys	113.00	4.00	118.00	7.31
Cysteic Acid	9.26	0.88	10.80	0.64
Thr	81.10	3.71	82.80	4.62
Ala	182.00	12.25	168.00	2.04
Met	28.06	2.31	23.10	1.09

^c^ All data is represented as mean ± SEM (*n* = 7–8). Dissimilar superscript letters represent significant differences (*p* < 0.05). Abbreviations: *HFD* 45 % energy high fat diet with 20 % energy casein, *HFD + Lac* 45 % energy high fat diet with 20 % energy bovine lactoferrin

### Dietary bovine lactoferrin moderately affected adipose gene expression

Given the reduction of plasma leptin concentrations in the HFD + Lac mice compared to the HFD group, leptin (*Lep*) mRNA levels were compared in adipose tissue samples. There were no significant changes between high fat diet fed mice beyond the significant increase found in both high fat diet fed groups compared to the LFD group (*P* < 0.05) (LFD: 1.00 ± 0.11; HFD: 3.29 ± 0.44; HFD + Lac: 3.69 ± 0.55).

To determine whether there were differences in mRNA levels of genes associated with the mechanisms surrounding lipid accretion given the reduced fat excretion in lactoferrin fed mice, we first examined CCAAT-enhancer-binding protein a (*Cebpa*), a gene associated with adipogenesis, and found a significant decrease in mRNA levels in the HFD + Lac and HFD group compared to the LFD mice (Additional file 3: Figure S1A). However protein expression of CEBPA p42 between groups was not altered (Additional file 4: Figure S2B). While no differences were found between high fat diet fed groups in the mRNA levels of β-oxidation related *Ppara* (Additional file 3: Figure S1B), western blot analysis indicated that SAT PPARA was significantly reduced in the HFD + Lac group compared to the LFD group, but not in the HFD group (Additional file 4: Figure S2A). No SAT alterations were found between the high-fat fed groups for genes associated with lipid uptake, specifically, lipoprotein lipase (*Lpl*), or genes associated with lipid catabolism namely, carnitine palmitoyltransferase 1b (*Cpt1b*) and beta-adrenergic receptor-3 (*b3ar*) (Additional file 3: Figure S1C-D). There was not a significant difference between high fat diet fed groups in mRNA level of peroxisome proliferator-activated receptor-gamma (*Pparg*), a gene associated with adipogenesis, nor was there a difference between high fat diet fed groups in the corticosterone activation associated gene, 11β-hydroxysteroid dehydrogenase type 1 (*Hsd11β1*) (Additional file 3: Figure S1E-F). The mRNA levels of glucocorticoid receptor (*Gccr*), associated with lipid uptake, were not significantly altered between groups (Additional file 3: Figure S1G). The SAT mRNA levels of genes associated with insulin signaling, namely insulin receptor (*Insr*) and glucose transporter 4 (*Glut4*) were also not significantly altered between high fat diet fed groups (data not shown).

In the EAT, *Lep* mRNA levels were not significantly altered between high fat diet groups (LFD: 1.00 ± 0.35; HFD: 2.82 ± 0.70; HFD + Lac: 3.65 ± 0.88). There were no significant differences between HFD + Lac and HFD groups in mRNA levels of *Cepba, Ppara* or *Lpl*, however there was a significant increase in the mRNA levels of *Cpt1b* in the HFD + Lac fed mice compared to the HFD group (Additional file 5: Figure S3A-D). Finally, no significant differences were found between groups in the mRNA levels of *Pparg, Hsd11β1, Gccr* (Additional file 5: Figure S3 E-G) or insulin related *Insr* and *Glut4* (data not shown).

## Discussion

The purpose of this study was to better understand the impact that dietary lactoferrin has on weight gain in comparison to dietary casein during a 13-week high fat dietary challenge. By the end of the trial, the HFD + Lac group showed no difference in bodyweight, weight gain, SAT mass, EAT mass, or % fat mass when compared to HFD fed mice. However, the lactoferrin fed mice did have significantly reduced plasma leptin concentrations that were not reflective of mRNA levels in either the SAT or EAT indicating that an impact on secretion or post-translational modification was responsible for this decrease. As plasma corticosterone levels were also reduced in the HFD + Lac group compared to the HFD fed group and leptin is linked with central control of the hypothalamus-pituitary-adrenal production of corticosterone, it is possible that the reduction in plasma leptin resulted in the reduction in plasma corticosterone levels, however, we found evidence that suggests that retinol metabolism in the hypothalamus may have also contributed to these effects.

The discrepancies between the findings in previous trials and the current one in relation to lactoferrin effects on weight gain and fat mass are likely due to variations in experimental method. For example, in the Shi et al*.* trial, the supplementation of lactoferrin, during weight loss and weight regain regimen may have facilitated lactoferrin to affect weight and fat mass [5]. In the Morishita et al. trial, which lasted 4 weeks, ICR mice were given lactoferrin through gastric intubation with a diet containing 10 % energy from fat. When the same trial was conducted in Sprague–Dawley rats fed a high fat diet, no beneficial change in visceral fat accumulation occurred [6]. The Morishita et al. study supported results from Takeuchi et al*.* that showed dietary bovine lactoferrin reduces plasma TAG and NEFA concentrations in mice fed a chow diet, however, when mice were fed a high fat diet, the benefits associated with lactoferrin were abolished [7]. Our results are in line with the Takeuchi and Morishita et al*.* studies [6].

Initially, mice fed the HFD + Lac averted significant weight gain compared to the LFD group until week 8, whereas, those fed casein gained significantly more weight than the LFD group by week 2. While previous studies have shown that mice fed a high fat diet with WPI and BSA have significantly reduced weight gain resulting in significantly reduced bodyweight over 13–14 weeks, the current study suggests that lactoferrin has a less potent effect on weight gain under similar conditions [3, 4]. We speculate that increased uptake of dietary fat in the small intestine may account for why initial reductions in weight gain did not extend past week 5 in the HFD + Lac group. This is based on the significant reduction of % fecal fat and the moderate increase in jejunal *Cd36/FAT* mRNA levels in the HFD + Lac group compared to the HFD fed mice.

Previously, Petit et al*.* reported that the gastrointestinal tract of adult mice fed a high fat diet for three weeks adapted to high levels of oral lipid intake through two gastrointestinal adaptations. The first of these, namely increased intestinal cell proliferation and increased villi height, led to an increase in absorptive area in the small intestine [16]. Presumably, the fat-mediated increase of intestinal absorptive area partially led to greater uptake of dietary fat in the Petit et al*.* study. Therefore, it is interesting that previous studies have shown that bovine lactoferrin also increases absorptive area in the gastrointestinal tract. Blais et al*.* reported that dietary bovine lactoferrin bound to lactoferrin receptors in the small intestine of adult mice leads to an increase in enterocyte proliferation, villus height, and reduction of cell apoptosis [17]. These findings could explain why HFD + Lac fed mice had reduced faecal fat content than HFD control.

The second gastrointestinal adaptation in response to high fat diet feeding is a fatty acid-induced increase in the expression of genes associated with lipid uptake, which included the long-chain fatty acid transporter *Cd36/FAT* [16]*.* Greater expression of CD36/FAT is thought to lead to greater efficiency in the uptake of lipids from the gastrointestinal lumen. This is consistent with the reduced fat excretion and the increase in CD36/FAT gene expression in the HFD + Lac, which only reached a trend towards significance from HFD. Interestingly, Petit et al*.* also reported that increases in intestinal Cd36/FAT expression leads to larger chylomicron size. Larger chylomicrons are hydrolysed faster by LPL and release more TAG into the blood [16], findings that are consistent with plasma TAG concentrations found in the HFD + Lac group when compared to the LFD group when the same significant differences between the HFD and LFD group were not present.

Despite similar weight gain to the HFD fed mice, HFD + Lac mice had significantly reduced circulating levels of leptin. It has been shown that ingested lactoferrin reduces visceral adipose tissue differentiation: however, this is only if lactoferrin is not digested by pepsin [6]. In support of previous findings, we did not see any changes in mRNA for genes associated with adipocyte differentiation between the HFD and HFD + Lac groups, namely *Pparg* and *C/ebpa*, nor were there differences between the two groups in adipose tissue mass or fat percentage. As reductions in fat mass could not explain the plasma leptin differences between the high fat diet groups, we examined SAT and EAT mRNA levels of *Lep*, the gene associated with leptin production. We found no changes between high fat diet groups in either adipose tissue indicating that posttranslational modification or mechanisms surrounding secretion contributed to the decrease of plasma leptin concentrations [18].

In search of the mechanisms underlying the presumed change in leptin secretion, we analysed plasma concentrations of amino acids and found a significant reduction of glutamate in the HFD + Lac mice. Glutamate is has been shown to be a powerful stimulant of leptin release in the absence of glucose or insulin [19]. Another significantly reduced amino acid in the HFD + Lac group was histidine. Histidine is a precursor of glutamate, therefore, it is interesting that both of these amino acids were reduced in the plasma of the HFD + Lac fed mice [19]. We also found that glycine, another amino acid associated with leptin release, was significantly increased in the HFD + Lac group compared to the HFD mice. Glycine acts to stimulate secretion of basal levels of leptin, however, glycine also prevents the ability of insulin to stimulate leptin secretion [19]. It is not known why these amino acid profiles were altered between the high fat diet groups, when metabolic activity was not significantly different. Further work is needed to identify the potential mechanisms involved.

In addition to reduced plasma leptin levels, the HFD + Lac group had significantly less plasma corticosterone than the HFD group. Leptin is linked with central control of the release of corticosterone, however it is unlikely that the reduction in leptin is responsible for the decreased corticosterone concentrations through *Crh* and *V1a* as neither were altered between groups in this study [20, 21]. Based on gene-set analysis, we speculate that the decrease in corticosterone occurred through the hypothalamic retinol metabolism pathway, which increased in the HFD + Lac group compared to the HFD group. This pathway ultimately leads to the production of retinol-based metabolites, such as 13-*cis*-retinoic acid and all-*trans*-retinoic acid [22]. Vitamin-A deficient rats have been shown to have HPA-axis hyperactivity in response to restraint stress without altering hypothalamic *Crh* [23]. However, retinoic acid also acts to increase hypothalamic *Crh* and *V1a* pathways and subsequently increases plasma corticosterone [22]. Thus, further work is needed to better understand what role, if any, the increase gene sets linked to retinol metabolism may have had in corticosterone metabolism in this study.

It is important to note that the gene set linked to feeding behaviour was down-regulated in the hypothalamus of HFD + Lac fed mice compared to HFD controls. The genes in that pathway included those associated with melanocortin signalling, which is positively regulated by leptin [24]. Notably, the decrease in the activity of this pathway was not reflected in energy intake or energy expenditure, presumably because of changes in other signalling pathways associated with energy balance, including retinoic acid signalling pathway and those regulated by corticosterone (see below).

What impact the reduction in plasma leptin and corticosterone levels played in the final fat mass accumulation of HFD + Lac fed mice remains to be determined. Excess amounts of glucocorticoids have been shown to increase the distribution of central adipose mass by increasing lipogenesis via *Gccr* and stimulation of *Lpl* expression [25]. It is noteworthy that mRNA levels of *Lpl* and *Gccr* were not altered between high fat diet groups. This coupled with the lower plasma corticosterone levels in the HFD + Lac fed mice with similar % fat mass to HFD controls suggests that reduced corticosterone signalling had no impact on lipid accumulation in the HFD + Lac group [26, 27].

In contrast, it is thought that the direct effect of leptin on lipid metabolism in vivo is minimal in comparison to the impact it has indirectly on energy intake [28]. Leptin’s regulation of insulin secretion and glucose metabolism are also thought to factor into lipid metabolism indirectly, and while plasma insulin was significantly increased in the HFD + Lac groups compared to the LFD, there were no differences in plasma glucose between HFD and HFD + Lac groups [28]. This indicates that the reduced leptin in the HFD + Lac group did not have a significant effect on lipid metabolism. Leptin increases cellular lipid metabolism via sympathetic catecholaminergic innervation of fat depots, which is specific to epididymal adipose tissue and leads to reduced *Pparg* and subsequently reduced adipogenesis [28]. However, EAT *Pparg* was not altered between high fat diet groups in this study. Collectively, the data suggest that the decrease in plasma leptin and corticosterone had little effect on adipose gene expression, which contrasts with the hypothalamus.

Finally, it is interesting to note the significant differences found between the HFD + Lac fed mice and the LFD fed mice not found between the HFD and LFD groups, because this suggests that a combination of dietary bovine lactoferrin and altered carbohydrate:fat ratio may have led to similar weight gain effects seen in the HFD but through different pathways. For example, leptin stimulates POMC neurons located in the arcuate nucleus (ARC) of the hypothalamus, which ultimately leads to decreased energy consumption and increased energy expenditure [29, 30]. The HFD + Lac group, which had significantly reduced plasma leptin, also had significantly reduced mRNA levels of hypothalamic *Pomc* compared to the LFD group. This is consistent with the finding that the HFD + Lac group consumed cumulatively more EI alongside a modest reduction in energy expenditure, as depicted by VO_2_ values, compared to the LFD group. No such reduction in VO_2_ values was found in the HFD group compared to the LFD fed mice. Potentially exasperating the effect of reduced circulating leptin on hypothalamic *Pomc* expression in the HFD + Lac group, orally administered bovine lactoferrin, once digested, has been shown to act as an opiod [31]. Opiods have been shown to reduce hypothalamic POMC expression via the opiodergic system [31, 32]. Therefore, HFD + Lac mice may have had similar fat mass and weight gain compared to the HFD due to a reduction in plasma leptin and subsequent reductions in hypothalamic POMC. Further work is needed to better understand what impact bovine lactoferrin as part of a diet high in fat reduced in carbohydrate has on weight gain, as it may ultimately lead to greater weight gain in the long term.

Further work is also needed to clarify why the HFD + Lac group took 8 weeks to become significantly greater in weight than the LFD group when it only took the HFD group 2 weeks. The limitations of the current study were that leptin and corticosterone plasma concentrations were only measured at the end of the trial. Ideally, both of these hormones should have been monitored throughout the study to identify a specific time point when leptin and corticosterone became reduced and what impact those reductions may have had on the final weight gain and bodyweight of mice fed a HFD + Lac.

## Conclusion

HFD + Lac fed mice gained similar amounts of weight gain and % fat to the mice fed HFD, but had significantly reduced plasma leptin possibly through reduced secretion, which can be linked to altered plasma amino acid profiles. The reduction in plasma leptin accompanied a decrease in expression of a hypothalamus associated gene set linked to feeding behaviour. Plasma corticosterone concentrations were significantly reduced in HFD + Lac fed mice, which could be associated with reduced expression of hypothalamic expression of the gene set linked to retinoic acid metabolism*.* Based on these results, lactoferrin in a diet-induced model of obesity, does not reduce weight gain or % fat mass better than casein over a prolonged period of time in male C57BL/6 J mice during adolescence to adulthood, however it may be useful as a means of further exploration into corticosterone and leptin pathways affecting hypothalamic gene expression without the confounding effects of body weight change.

## Additional files

Additional file 1: Table S1. Diet composition. (PDF 104 kb) Additional file 2: Table S2. Sequences of primers^1^ used in gene expression analysis. (PDF 13 kb) Additional file 3:
**Subcutaneous adipose tissue mRNA level analysis.** (TIF 109 kb) Additional file 4:
**Western blot analysis of subcutaneous CEBPA and PPARA.** (TIF 234 kb) Additional file 5:
**Epididymal adipose tissue mRNA level analysis.** (TIF 112 kb)

## Abbreviations

EE: Energy expenditure
EI: Energy intake
HFD: High fat diet with casein
HFD + Lac: High fat diet with lactoferrin
LFD: Low fat diet with casein
RER: Respiratory exchange ratio
TAG: Triacylglycerol
VCO_2_: Carbon dioxide production
VO_2_: Oxygen consumption
