# Supplementary material for: Compared to casein, bovine lactoferrin reduces plasma leptin and corticosterone and affects hypothalamic gene expression without altering weight gain or fat mass in high fat diet fed C57/BL6J mice
Source: Nutr Metab (Lond). 2015 Dec 8;12:53. doi: 10.1186/s12986-015-0049-7 (PMC4672495; doi:10.1186/s12986-015-0049-7)
Supplement: Additional file 1: Table S1. — Diet composition. (PDF 104 kb) [file 12986_2015_49_MOESM1_ESM.pdf]

Supplemental Table 1. Diet composition

	(LFD) D12450B		(HFD) D12451		(HFD+Lac) D12011802	
%	g	kcal	g	kcal	g	kcal
Protein	19.2	20	23.7	20	23.7	20
Carbohydrate	67.3	70	41.4	35	41.4	35
Fat	4.3	10	23.6	45	23.6	45
Total		100		100		100
kcal/g	3.8		4.7		4.7	
Ingredients	g	kcal	g	kcal	g	kcal
Casein	200	800	200	800	0	0
Lactoferrin	0	0	0	0	200	800
L-Cystine	3	12	3	12	3	12
Corn Starch	315	1260	72.8	291	72.8	291
Maltodextrin 10	35	140	100	400	100	400
Sucrose	350	1400	172.8	691	172.8	691
Cellulose, BW200	50	0	50	0	50	0
Soybean oil	25	225	25	225	25	225
Lard	20	180	177.5	1598	177.5	1598
Mineral Mix S10026	10	0	10	0	10	0
DiCalcium Phosphate	13	0	13	0	13	0
Calcium Carbonate	5.5	0	5.5	0	5.5	0
Potassium Citrate, 1 H_20	16.5	0	16.5	0	16.5	0
Vitamin Mix V10001	10	40	10	40	10	40
Choline Bitartrate	2	0	2	0	2	0
FD&C Yellow Dye 5	0.05	0	0	0	0	0
FD&C Red Dye 40	0	0	0.05	0	0	0
FD&C Blue Dye 1	0	0	0	0	0.05	0
Total	1055.05	4057	858.15	4057	858.15	4057

Abbreviations: LFD, 10% energy fat, 20% energy casein; HFD, 45% energy fat, 20% energy casein; HFD+Lac, 45% energy fat, 20% energy bovine lactoferrin
