# Supplementary material for: Compared to casein, bovine lactoferrin reduces plasma leptin and corticosterone and affects hypothalamic gene expression without altering weight gain or fat mass in high fat diet fed C57/BL6J mice
Source: Nutr Metab (Lond). 2015 Dec 8;12:53. doi: 10.1186/s12986-015-0049-7 (PMC4672495; doi:10.1186/s12986-015-0049-7)
Supplement: Additional file 2: Table S2. — Sequences of primers1 used in gene expression analysis. (PDF 13 kb) [file 12986_2015_49_MOESM2_ESM.pdf]

Supplementary Table 2. Sequences of primers[1] used in gene expression analysis

Gene	Forward Primer (5'-3')	Reverse Primer (5'-3')
B3AR	5'- CGCCTTCAACCCGGTCATCTACTG -3'	5'- GGTGGACTCTGCCTGGCTTCAAC -3'
CEBPA	5'- GGGTGGCGGCGGTGACTTTGACTAC -3'	5'- CGCGCTCGTACAGGGGCTCCAG -3'
CEBPD	5'- TCGCCGACCTCTTCAACAGCAACC -3'	5'- GTCGCCGTCGCCCCAGTCG -3'
CD36	5'- CCGGTGTGGTGGCTGCTCTTCTC -3'	5'- GCTGCCATCTCCCCGCCATAAATG -3'
CPT1B	5'- CGAGAGGGGCGGACTGAGACTG -3'	5'- GGCTAGGCGGTACATGTTTTGGTG -3'
CRH	5'-GGAAATGGCCCGGGCAGAGCAG-3'	5'-TGGCCAAGCGCAACATTTCATTTC-3'
FASN	5'- TCCACCTTTAAGTTGCCCTG -3'	5'- TCTGCTCTCGTCATGTCACC -3'
FATP1	5'- CCGGTGTGGTGGCTGCTCTTCTC -3'	5'- GCTGCCATCTCCCCGCCATAAATG -3'
FGF21	5'-AGCGCAGCCCTGATG-3'	5'-TCAGGGATGGGGTAT-3'
GCCR	5'- ACCTGACTTCCTTGGGGGCTATGA -3'	5'- CAGGCAGAGTTTGGGAGGTGGTC -3'
GLUT4	5'- GGCCTGCCCGAAAGAGTC -3'	5'- AGGAGCTGGAGCAAGGAC -3'
HSD11B1	5'- CCTTGGCTGGGAAAATGACC -3'	5'- CTATGAGGCCAAGGACACAGAGAG -3'
INSR	5'- GATTTCCCCAACGTCTCCTCTAC -3'	5'- CAATGCGGTACCCAGTGAAGTG -3'
IRS1	5'- GCGCAGGCACCATCTCAACAACC -3'	5'- GCACGCACCCGGAAGGAACC -3'
LEP	5'- CCCCGCACCGCTGGAAGTAC -3'	5'- ATGTGCCCTGAAATGCGGTAT -3'
LPL	5'- TGCTCCCAACAATATAAGACTCC -3'	5'- AAGGCCAGGTGTTTCAATC -3'
NPY	5'- CTCCGCTCTGCGACACTACA -3'	5'- AATCAGTGTCTCAGGGCTGGA -3'
ObR	5'- GACCACCGAACACAACCGATGAC -3'	5'- ACACCTAGCTGGCGAAAAACTGAAG -3'
PPARA	5'- ATGGGGGTGATCGGAGGCTAATAG -3'	5'- GGGTGGCAGGAAGGGAACAGAC -3'
PPARG	5'- TCAGGTTTGGGCGGATGC -3'	5'- TCAGCGGGAAGGACTTTATGTATG -3'
POMC	5'- GGGCAAGCGCTCCTACTCCATG -3'	5'- CTCGCCTTCCAGCTCCCTCTTG -3'
UCP3	5'- ACAGGCCCACACGGTCCAGAACC -3'	5'- CCCATCAGGTCAGTGCAAAACAGAGG -3'
V1A	5'-CTAACAACCGAAGCCCAACAAAC-3'	5'-ACACAAGCCCCGTCCAAGAGTC-3'
Reference	Genes	
18-S	5'- AGGACCGCGGTTCTATTTTGTTGG -3'	5'- ATGCTTTCGCTCTGGTCCGTCTTG -3'
ACTB	5'- AGAGGGAAATCGTGCGTGAC -3'	5'- CAATAGTGATGACCTGGCCGT -3'
YWHAZ	5'- CGGAGCTGCGTGACATCTGC -3'	5'- CCTCGGCCAAGTAACGGTAGTAG -3'

[1] B3AR, Beta-3 Adrenergic Receptor; CEBPA/D, CCAAT/Enhancer Binding Protein alpha/delta; CD36, fatty acid translocase CPT1/B, Carnitine Palmitoyltransferase B; CRH, corticosterone hormone; FABP1, Fatty Acid Binding Protein 1; FASN, Fatty Acid Synthase; FATP1, Fatty Acid Transport Protein 1; FGF21, fibroblast growth factor; GCCR, Glucocorticoid Receptor; GLUT4, glucose transporter 4; HSD11B1, Hydroxysteroid (11-beta) Dehydrogenase 1; INSR, insulin receptor; IRS1, insulin receptor substrate 1; LEP, Leptin; LPL, Lipoprotein Lipase; NPY, Neuropeptide Y; ObR, Leptin Receptor; PPARA/G, Peroxisome Proliferator-Activated Receptor alpha/gamma; POMC, Pro-opiomelanocortin; UCP 3, Uncoupling Protein 3; V1A, arginine vasopressin receptor 1a

18-S, 18-S ribosomal RNA; ACTB, Beta Actin; YWHAZ, Tyrosine 3-monooxigenase/ Tryptophan 5-monooxigenase Activation Protein Zeta Polypeptide
