# Supplementary figures and images for: Compared to casein, bovine lactoferrin reduces plasma leptin and corticosterone and affects hypothalamic gene expression without altering weight gain or fat mass in high fat diet fed C57/BL6J mice
Source: Nutr Metab (Lond). 2015 Dec 8;12:53. doi: 10.1186/s12986-015-0049-7 (PMC4672495; doi:10.1186/s12986-015-0049-7)

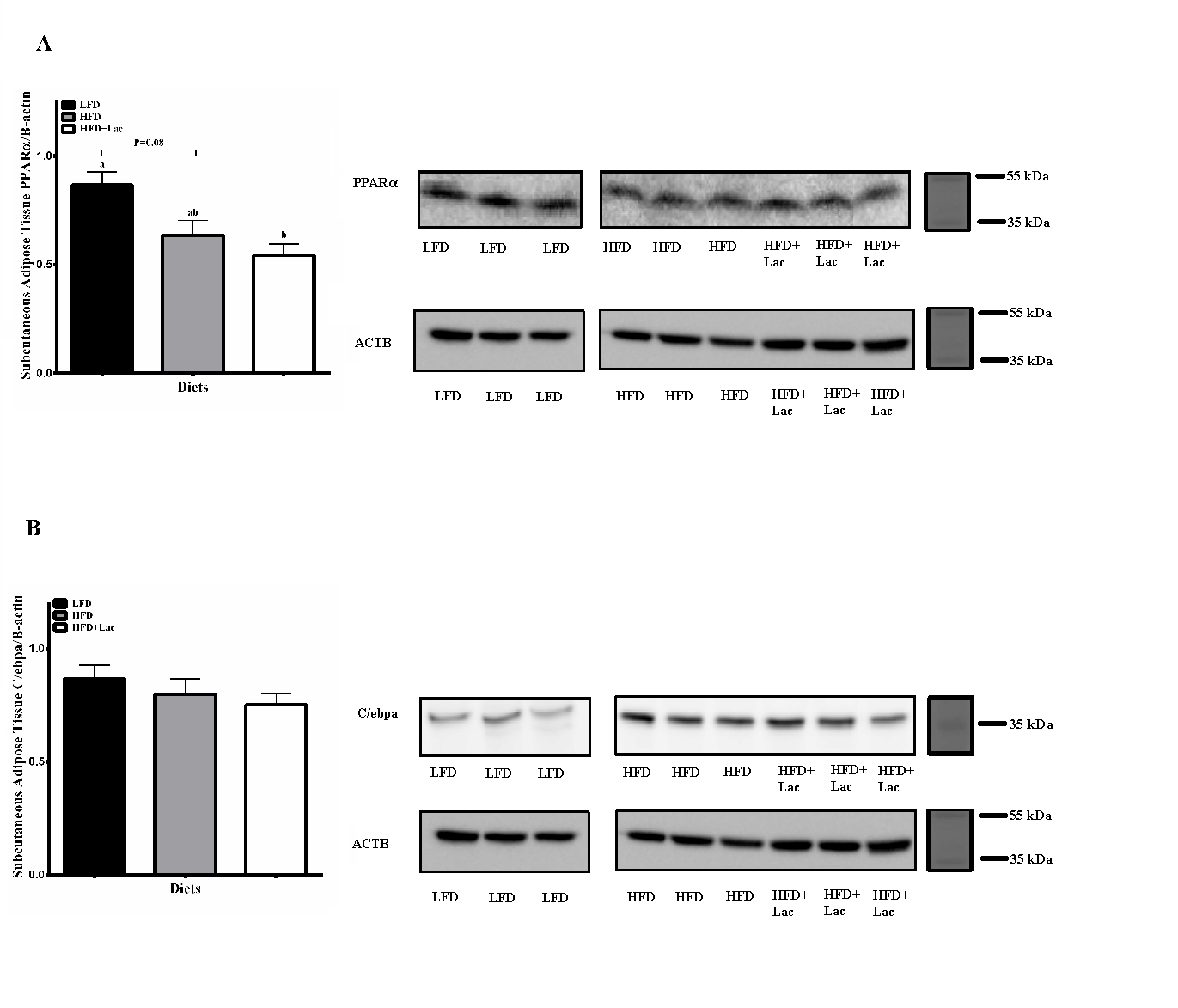

Supplement: Additional file 4: — Western blot analysis of subcutaneous CEBPA and PPARA. (TIF 234 kb) [file 12986_2015_49_MOESM4_ESM.tif]
